# MMP-2/9-Specific Activatable Lifetime Imaging Agent

**DOI:** 10.3390/s150511076

**Published:** 2015-05-12

**Authors:** Marcus T.M. Rood, Marcel Raspe, Jan Bart ten Hove, Kees Jalink, Aldrik H. Velders, Fijs W.B. van Leeuwen

**Affiliations:** 1 Interventional Molecular Imaging Laboratory, Department of Radiology, Leiden University Medical Center, Leiden 2300RC, The Netherlands; E-Mails: m.t.m.rood@lumc.nl (M.T.M.R.); janbart.tenhove@wur.nl (J.B.H.); aldrik.velders@wur.nl (A.H.V.); 2 Division of Cell Biology I, Netherlands Cancer Institute, Amsterdam 1066CX, The Netherlands; E-Mails: m.raspe@nki.nl (M.R.); k.jalink@nki.nl (K.J.); 3 Laboratory of BioNanoTechnology, Wageningen University, Wageningen 6700EK, The Netherlands

**Keywords:** iridium, lifetime imaging, luminescence, enzymatic activation, MMP, FRET, fluorescence

## Abstract

Optical (molecular) imaging can benefit from a combination of the high signal-to-background ratio of activatable fluorescence imaging with the high specificity of luminescence lifetime imaging. To allow for this combination, both imaging techniques were integrated in a single imaging agent, a so-called activatable lifetime imaging agent. Important in the design of this imaging agent is the use of two luminophores that are tethered by a specific peptide with a hairpin-motive that ensured close proximity of the two while also having a specific amino acid sequence available for enzymatic cleavage by tumor-related MMP-2/9. Ir(ppy)_3_ and Cy5 were used because in close proximity the emission intensities of both luminophores were quenched and the influence of Cy5 shortens the Ir(ppy)_3_ luminescence lifetime from 98 ns to 30 ns. Upon cleavage *in vitro,* both effects are undone, yielding an increase in Ir(ppy)_3_ and Cy5 luminescence and a restoration of Ir(ppy)_3_ luminescence lifetime to 94 ns. As a reference for the luminescence activation, a similar imaging agent with the more common Cy3-Cy5 fluorophore pair was used. Our findings underline that the combination of enzymatic signal activation with lifetime imaging is possible and that it provides a promising method in the design of future disease specific imaging agents.

## 1. Introduction

The use of luminescence in molecular imaging has many advantages. Luminescence is a high-resolution, real-time imaging technology that requires low concentrations of label. However, there are also disadvantages. Two main disadvantages of luminescence imaging are the presence of autofluorescence and background signal as a result from non-specific retention of an imaging agent [1].

Autofluorescence is the combined name for all emissions by endogenous compounds, and is mainly associated with molecules such as NADH and flavins [2]. Although the emission intensity of such endogenous molecules is generally weaker than that of specifically designed luminophores, these emissions can still obscure imaging findings [3]. Luminescence lifetime, a parameter that describes the time between excitation and signal emission, offers a technology that can be exploited to separate luminescent signals of different origins. Most endogenous and organic luminophores have luminescence lifetimes in the same range (0.1–10 ns) [4]. To reduce the background caused by endogenous emissions, dedicated lifetime imaging agents should have a lifetime that lies well beyond this range [5]. A luminescence lifetime of 20 ns can already easily be separated from shorter lifetimes using lifetime gating [6]. There is also a downside to lifetime elongation, since the use of longer luminescence lifetimes decreases imaging speed and photon flux. This is important if small volumes and low concentrations are measured, for instance in confocal microscopy, and also when real-time images are required, like in image-guided surgery. To obtain high-quality imaging that is fast enough for these applications, a maximum lifetime of 100 ns has been estimated [7]. Such luminescence lifetimes cannot be obtained using organic luminophores but are typical for transition metal ion complexes based on, e.g., ruthenium (II) or iridium (III) [8,9,10]. 

The presence of non-specifically bound imaging agent increases background signal and thereby reduces imaging quality. Imaging agents can for instance remain present in blood, or give non-specific uptake in tissue/organs [11]. In luminescence-based molecular imaging, this disadvantage can be overcome by disease-related enzyme-controlled activation of a luminescent signal; before activation, the signal should be in the off-state (quenched) [12]. The most promising enzymes in this application are the ones that are associated with the presence of malignant tissue, such as cathepsins [13], gamma-glutamyl transpeptidase [14] and matrix metalloproteinases (MMP) [15,16]. Many different photophysical and chemical quenching mechanisms have been studied for activatable luminescent imaging agents, including Förster Resonance Energy Tranfer (FRET) [17,18,19], photon-induced electron transfer [20,21], chemical reactions on the luminophore itself [22], and spin-orbit coupling [23]. In theory, for each enzyme that can cleave a chemical bond, a suitable activatable imaging agent can be generated to ensure local and specific signal enhancement [24]. 

MMP-2 and MMP-9 are closely related and they both cleave the same PLGLA peptide sequence [15,25]. MMP-2/9 have the natural function of cleaving the extracellular matrix, which is rarely required in healthy tissue, but since tissue reconstruction is needed around fast-growing tumors, the presence of MMP-2/9 is said to be related to the presence of invasive tumor tissue [26,27]. The family of MMPs has proven its potential in oncology related molecular imaging, with applications extending as far as fluorescence-guided surgery [28].

We have previously shown that covalent attachment of Ir(ppy)_3_ and Cy5 using a disulfide bond generates a luminophore pair in which a combination of spin-orbit coupling by the iridium atom and FRET quenches the luminescent signal of both luminophores [29]. This quenching was accompanied by a shortening of the Ir(ppy)_3_ related lifetime. Cleavage of the connective disulfide bond led to the restoration of the luminescence intensity of both luminophores and an elongation of the lifetime of the Ir(ppy)_3_ emission [29]. In this approach, the luminescence lifetime should allow for the separation of luminescence signal from autofluorescence, while the signal activation should reduce the background signal generated by non-specifically distributed imaging agent [29,30,31]. We therefore reasoned that a Ir(ppy)_3_-Cy5 pair combined with MMP-2/9 selective signal activation has the potential to provide a new generation of molecular imaging agents. 

## 2. Experimental Section 

### 2.1. General

All chemicals were obtained from commercial sources and used without further purification. HPLC was performed on a Waters system by using a 1525EF pump and a 2489 UV detector. For preparative HPLC a Dr. Maisch, GmbH, Reprosil-Pur 120 C18-AQ 10 μm (250 × 20 mm) column was used and a gradient of 0.1% TFA in H_2_O/CH_3_CN (95:5) to 0.1% TFA in H_2_O/CH_3_CN (5:95) in 40 min was employed. For analytical HPLC a Dr. Maisch, GmbH, Reprosil-Pur C18-AQ 5 μm (250 × 4.6 mm) column was used and a gradient of 0.1% TFA in H_2_O/CH_3_CN (95:5) to 0.1% TFA in H_2_O/CH_3_CN (5:95) in 40 min was employed. HPLC traces of the final compounds are included in the Supplementary Materials, conforming >95% purity. MALDI-ToF measurements were performed on a Bruker Microflex system.

### 2.2. Synthesis of Peptide-Cy5 (**1**)

Two very similar peptides were synthesized on Rink Amide Resin using standard Fmoc-strategy solid phase peptide synthesis methods. The sequences were as follows:

$$\begin{array}{l} {\text{NH}}_{2}\text{-eeeeeGPLGLArrrrrrrrk-RESIN (L-amino acids)}\\ {\text{NH}}_{2}\text{-eeeeegplglarrrrrrrrk-RESIN (D-amino acids)} \end{array}$$

**Scheme 1:**
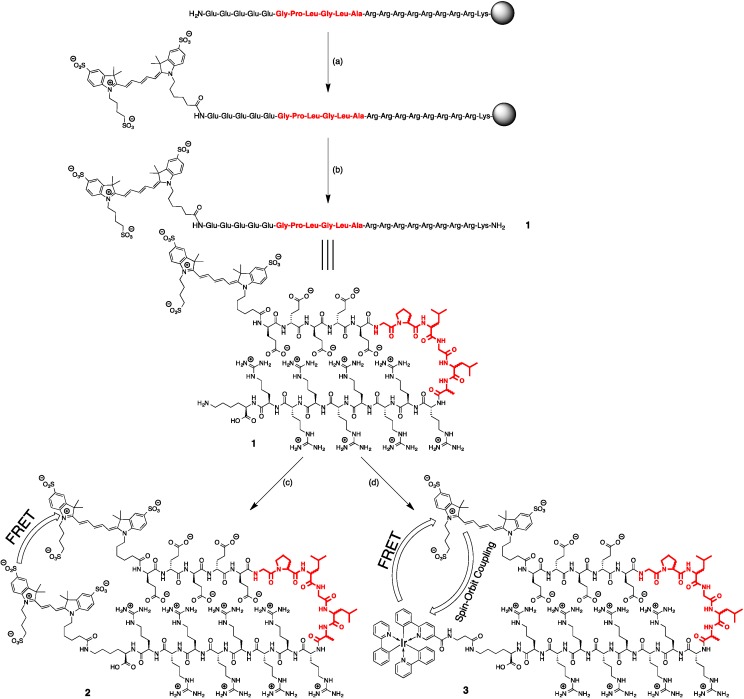
Synthesis of the activatable imaging agents discussed in this research. (**a**) Cy5, PyBOP, DIPEA, DMF; (**b**) TFA, H_2_O, TIS; (**c**) Cy3-NHS, H_2_O/DMSO; (**d**) Ir(ppy)_3_-β-Ala-COOH, DCC, NHS, H_2_O/DMSO. The amino acid sequence for cleavage is highlighted in red and comprised of either l-amino acid or d-amino acids. All other amino acids used to generate these structures were d-amino acids.

The only difference between the two peptides was the stereochemistry of the six amino acids of the cleavage site (highlighted in red in Scheme 1). Both peptides were treated equally for the next steps. Cy5-COOH was coupled to the peptide using standard protocol for solid phase labeling. In short, to the resin-bound peptide with all protecting groups attached (10 μmol) in DMF (2 mL) was added Cy5 (17 mg, 20 μmol), PyBOP (10.4 mg, 20 μmol) and DIPEA (6.8 μL, 40 μmol). The reaction was stirred overnight at RT, after which the resin was washed three times with DMF and three times with DCM. After drying, the dark-blue resin in vacuum, the peptide was cleaved from the resin using 1 mL cleavage mixture (92.5% TFA: 5% H_2_O: 2.5% TIS) for 4 h at RT. The solution was collected and the resin was washed with TFA until the solution became colorless. The TFA solution was added dropwise to MTBE:hexane 1:1 at −20 °C to give a blue precipitation. The blue precipitation was washed with MTBE:hexane 1:1, dissolved in H_2_O and lyophilized to give a blue solid.

For **1L**, the yield was 27.4 mg (87%). m/z (MALDI-ToF): [C_138_H_231_N_48_O_40_S_3_]^+^ calcd 3298.9, found 3298.4. For **1D**, the yield was 12.0 mg (36%). m/z (MALDI-ToF): [C_138_H_231_N_48_O_40_S_3_]^+^ calcd 3298.9, found 3298.9. 

### 2.3. Synthesis of Cy3-Cy5 Peptides (**2L** and **2D**)

**1L** (1.4 mg, 0.42 μmol) was dissolved in 4 mL dry DMSO and DIPEA (5 μL, 28 μmol) was added. Cy3-NHS (1 mg, 1.2 μmol) was dissolved in 100 μL dry DMSO and added to the reaction mixture. The mixture was stirred at RT for 7 days, after which the product was purified by HPLC. The product peak was collected and lyophilized to give a purple solid (**2L**, 0.3 mg, 18%). *m*/*z* (MALDI-ToF): [C_171_H_271_N_50_O_50_S_6_]^+^ calcd 4019.7, found 4020.2.

**1D** (0.6 mg, 0.19 μmol) was dissolved in 1 mL DMSO and 2 mL 0.1 M phosphate buffer pH 8.0 (no further base was added since this would increase the likelihood of hydrolysis). Cy3-NHS (0.8 mg, 1.0 μmol) was dissolved in dry DMSO added to the reaction mixture. Work-up was similar to **2L** to give **2D** (0.3 mg, 39%). *m*/*z* (MALDI-ToF): [C_171_H_271_N_50_O_50_S_6_]^+^ calcd 4019.7, found 4020.7.

### 2.4. Synthesis of Ir(ppy)_3_-Cy5 Peptides (**3L** and **3D**)

Ir(ppy)_3_-COOH (5.4 mg, 7 μmol), synthesized as previously described [32], was dissolved in 500 μL DMSO. To this solution was added NHS (1.2 mg, 10 μmol) and DCC (1.2 mg, 6 μmol) and the reaction was stirred for 15 min at RT to form Ir(ppy)_3_-NHS *in situ*.

Three hundred microlitres of the freshly made solution of Ir(ppy)_3_-NHS (4.2 μmol) was added to a solution of **2** (**L**: 5.1 mg, 1.5 μmol; **D**: 1.4 mg, 0.4 μmol) in 500 μL phosphate buffer (pH 8.0). The solution was stirred overnight at RT, and a precipitation formed. This precipitate was redissolved and purified by HPLC. The product peak was collected and lyophilized to give a blue-green solid. The yield of **3L** was determined by UV/Vis to be 5.1 nmol (<1%). *m*/*z* (MALDI-ToF): [C_175_H_258_IrN_52_O_42_S_3_]^+^ calcd 4050.7, found 4052.4. The yield of **3D** was 3.8 nmol (<1%). *m*/*z* (MALDI-ToF): [C_175_H_258_IrN_52_O_42_S_3_] calcd 4050.7, found 4047.8.

### 2.5. Spectroscopic Measurements

All spectroscopic measurements were performed in quartz cuvettes. UV-Vis measurements were performed on an Amersham Ultrospec 3000. Emission spectra were measured using a Perkin Elmer S55. For fluorescence measurements, the absorbance was kept below 0.1 to prevent inner filter effects. Luminescence lifetime measurements were carried out using time-correlated single photon counting on a F900 Spectrometer (Edinburgh Instruments, Livingston, UK). For this, Ir(ppy)_3_ compounds (**3L** and **3D**) were dissolved in phosphate buffered saline (PBS) and excited using a picosecond pulsed diode laser (PicoQuant LDH-P-C-375, 372 nm) operated at 2.5 MHz. Luminescence emission was detected at 600 nm on a microchannel plate detector. Lifetimes were fit to a biexponential decay model using one long lifetime of 100 ns and one short lifetime of 1.0 ns.

### 2.6. Calculation of Distances between Luminophores

Distances between Cy3 and Cy5 were estimated using Equation (1) [33]. Peak intensities at 566 nm were used, and *R*_0_ was set as 53 Å [34].

(1) $$1-\frac{F_{DA}}{F_{D}}=\;\frac{R_{0}^{6}}{R_{0}^{6}+r^{6}}$$

*F_DA_* = Fluorescence intensity of donor in the presence of acceptor; *F_D_* = Fluorescence intensity of donor emission in the absence of acceptor; *R*_0_ Forster radius; *r* = distance between donor and acceptor.

Distances between Ir(ppy)_3_ and Cy5 were estimated using Equation (2) [33], and *R*_0_ was set as 48 Å [29].

(2) $$1-\frac{\tau_{DA}}{\tau_{D}}=\;\frac{R_{0}^{6}}{R_{0}^{6}+r^{6}}$$

τ*_DA_* = Luminescence lifetime of donor in the presence of acceptor; τ*_D_* = Luminescence lifetime of donor in the absence of acceptor; *R*_0_ Forster radius; *r* = distance between donor and acceptor.

### 2.7. Enzymatic Peptide Cleavage by Cells in Suspension

The individual imaging agents (**2L**, **2D**, **3L**, **3D**) were dissolved in 2 mL buffer (100 mM NaCl, 10 mM CaCl_2_, 50 μM ZnCl_2_, 50 mM Tris, pH 7.1) in a quartz cuvette to create a 0.2 μM solution. To this 2.0 × 10^4^ SKOV-3 cells in 500 μL DMEM were added. Enzymatic assays were performed at 37 °C to mimic physiological conditions as much as possible. Fluorescence spectra of the compounds were measured with intervals of 1–2 h up to 24 h to monitor the cleavage process.

For **2L** and **2D**, spectra were measured by excitation at 525 nm (Cy3 excitation), while the emission was detected between 540 and 750 nm. For **3L** and **3D**, Ir(ppy)_3_ spectra were measured by excitation at 450 nm, and emission detection was measured between 480 and 750 nm. Cy5 spectra (**3L**, **3D**) were measured using excitation at 625 nm, and emission detection between 640 and 750 nm. 

### 2.8. Enzymatic Peptide Cleavage by Adherent Cells

The MMP-2/9 expressing human ovary cancer cell line SKOV-3 [35] was maintained in Dulbecco’s minimum essential medium (DMEM) enriched with 10% fetal bovine serum and 5 mL Penicillin/Streptomycin (10,000 units/mL Penicillin; 10,000 μg/mL Streptomycin) (all Life Technologies Inc., Breda, The Netherlands). Cells were kept under standard culture conditions. The cells were grown on a glass-bottom well, after which they were incubated at 37 °C in a solution of an activatable imaging agent in DMEM for 24 h (0.2 μM for **2L** and **2D**; 0.5 μM for **3L** and **3D**). After incubation, cells were thoroughly washed with PBS. No negative effects of the compounds on cell growth were observed.

### 2.9. Confocal Imaging

After incubation, live cell images were taken on a Leica SP5 confocal microscope under 63× magnification. Ir(ppy)_3_ luminescence was measured using 458 nm excitation (Ar laser line) and emission was collected between 560 and 620 nm. Cy3 fluorescence was measured using 514 nm excitation (Ar laser line) and the emission was collected between 550 and 600 nm. Cy5 fluorescence was measured using excitation at 633 nm (HeNe laser) and the emission was collected between 650 and 700 nm.

### 2.10. Fluorescence Lifetime Imaging Microscopy

FD-FLIM images of SKOV-3 cells incubated with **3L** or **3D** (0.5 μM, 24 h) were obtained with LI-FLIM hardware (Lambert Instruments, Roden, The Netherlands) and software (v.1.2.23.59) with a modulated GaAs intensifier fiber-optically coupled to a digital CCD-camera (LI^2^CAM, Lambert Instruments) attached to the microscope (Leica DMIRE2; Leica Microsystems, Heidelberg, Germany) with a 63× objective (numerical aperture 1.3, glycerine-immersion). A 1-W, 442-nm LED was modulated at 1 MHz, and light passed a CFP/YFP filtercube (excitation 436/12 & 500/20, dichroic 445 & 515, emission 467/37 & 545/45). Rhodamine 6G in H_2_O was used as reference (τ_phase_: 3.83 ns at 37 °C) (Sigma-Aldrich, St. Louis, MO, USA). The cells were placed in preheated (37 °C) 4-(2-hydroxyethyl)-1-piperazineethane-sulfonic acid (HEPES)–buffered saline (140 mM NaCl, 5 mM KCl, 1 mM MgCl_2_, 1 mM CaCl_2_, 10 mM glucose, and 10 mM HEPES, pH 7.4) after washing with PBS. All experiments were performed at 37 °C.

## 3. Results and Discussion

### 3.1. Design and Synthesis of the Imaging Agents

**Figure 1:**
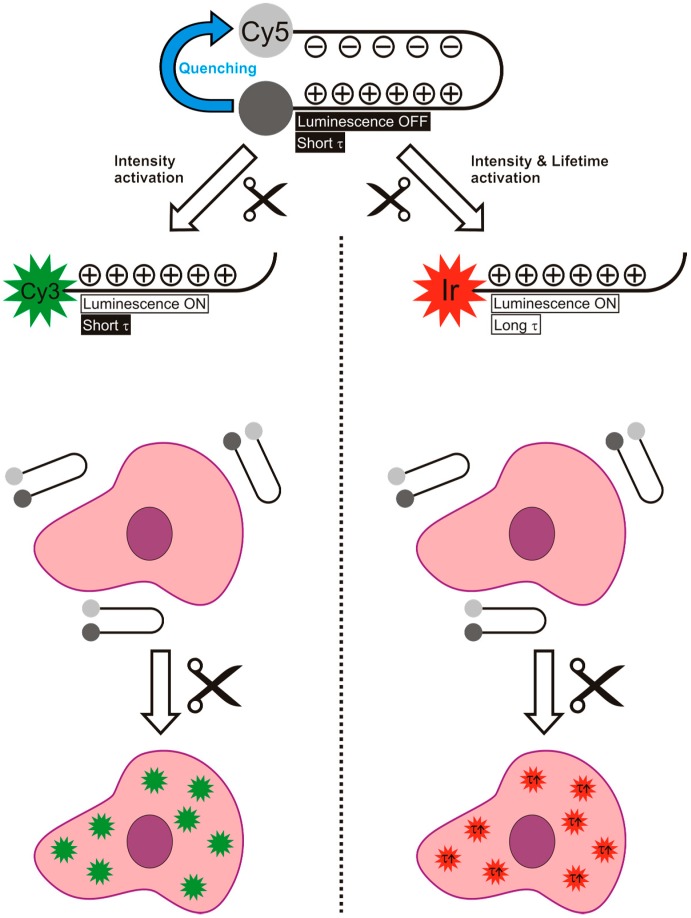
Schematic overview of the difference between the use of an activatable luminescence imaging agent (**left**) and an activatable lifetime imaging agents (**right**), both based on a hairpin motive. It is important to note that in the case of lifetime activation not only the intensity increases, but also the lifetime. This gives two parameters to follow the activation reaction.

Use of a hairpin motive is a known method to bring two luminophores within close proximity of each other and has already proven to be of value for the detection of thrombin [36] and MMP-2/9 [37]. In the hairpin motive used in our study, the electrostatic interactions between the positively charged arginine residues and the negatively charged glutamic acid residues ensure conformational rigidity, while leaving the cleavage site freely available for the MMP-2/9 enzymes (Figure 1) [15]. To prevent non-specific cleavage (e.g., by other peptidases), except for the cleavage site, the hairpin motive was generated out of d-amino acids.

The synthesis of all agents was successful. Labeling of the D & L hairpin motive peptides with Cy5 resulted in yields of 87% (**1L**) and 36% (**1D**). Generation of the reference compounds containing Cy3 and Cy5 gave reasonable yields of 18% (**2L**) and 39% (**2D**). The relatively poor solubility of **1** in DMSO proved to be limiting for the reaction to occur. In fact, for the synthesis of **2D**, the solvent of the reaction was changed to a water/DMSO mixture, despite the risk of hydrolyzing Cy3-NHS. It proved to be more difficult to obtain the imaging agents containing Ir(ppy)_3_ and Cy5, **3L** and **3D**, resulting in very low yields of <1%, which was, however, enough to perform proof-of-concept studies. Ir(ppy)_3_-NHS had to be synthesized *in situ* and directly used, since the activated ester was not soluble in water and its purification was cumbersome. As one may realize, this increased the solubility issues even further. To still allow for a reaction to take place, Ir(ppy)_3_-NHS (in DMSO) had to be added quickly to a stirring solution of **1** in aqueous solution (phosphate buffer pH 8.0). These non-ideal conditions resulted in precipitation of compounds and a large amount of hydrolysis of the active ester. Given our focus on the utility of the compounds generated, no further attempts were made to optimize the yields.

### 3.2. Photophysical Analysis of the Imaging Agents 

**Figure 2:**
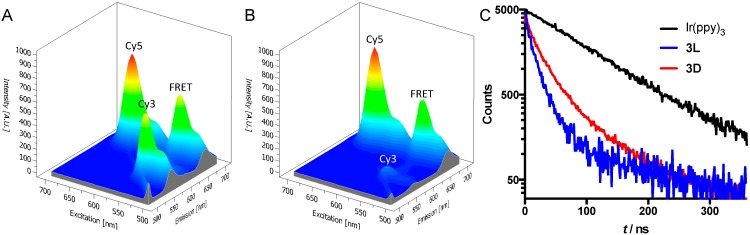
(**A**,**B**) Excitation-emission plots of peptides **2L** (**A**) and **2D** (**B**). The peaks labeled as “FRET” are the peaks that show acceptor emission (670 nm) with donor excitation (550 nm); (**C**) Luminescence decay traces at 600 nm of **3L**, **3D**, and reference compound Ir(ppy)_3_-COOH in water. All compounds were excited with a 372 nm laser at 2.5 MHz.

In the reference compounds **2L** and **2D**, the close proximity of the donor (Cy3) and acceptor (Cy5) yielded a very clear FRET interaction, as expected [34,38]. In the excitation-emission plots, a peak that corresponds to donor excitation (550 nm) and acceptor emission (670 nm) can be seen for both compounds (Figure 2A,B). However, there are clear differences in quenching efficiency between **2L** (43% quenching) and **2D** (88% quenching) (Figure 2). Based on the fluorescence intensities, we calculated the Cy3-Cy5 distances to be 53 Å for **2L** and 38 Å for **2D**. This difference in calculated distance between the dyes can be caused by a slightly different structural configuration of the hairpin structure with the D-amino acids compared to the one with L-amino acids. Since FRET efficiency depends to the sixth power on the donor-acceptor distance, these relatively small differences in the hairpin configuration may explain the much larger differences in FRET efficiency. Another possibility is that the difference in amino acid stereochemistry leads to a difference in dipole orientation. This also has a pronounced effect on the correct calculation of the distances [33].

For peptides **3L** and **3D**, no FRET signals could be detected using the spectrometer. In fact, emissions of both Ir(ppy)_3_ and Cy5 were efficiently quenched. This indicates that the earlier-described double quenching interactions between Cy5 and Ir(ppy)_3_ are also applicable in this hairpin structure [25,29]. In short: emission from Ir(ppy)_3_ is quenched by FRET to Cy5, while the Ir atom causes quenching of Cy5 emission. The emission of Cy5 was quenched very efficiently in peptides **3L** and **3D**, with a quenching efficiency of 91%–92% for both compounds compared to pure Cy5. The average lifetime of Ir(ppy)_3_emission in the peptides (30 ns for **3L** and 43 ns for **3D**) was measured to be clearly different from that of pure Ir(ppy)_3_-COOH (98 ns, Figure 2C). This reduction of lifetime of Ir(ppy)_3_ is indicative for a quenching interaction between both luminophores [33]. 

Ir(ppy)_3_-Cy5 distance calculations based on emission intensities proved to be inaccurate due to the low signal intensities. Instead, donor-acceptor distances were estimated based on the luminescence lifetime [33]. This gave distances between Ir(ppy)_3_ and Cy5 of 42 Å for **3L** and 46 Å for **3D**. These distances are in the same order as the distances estimated for **2L** and **2D**. Although these distances are generally considered to be too long for efficient spin-orbit coupling [39], still efficient Cy5 quenching was observed. This might indicate that a different quenching mechanism could be responsible for the quenching of Cy5 by Ir(ppy)_3_; potential candidates include static quenching, excimer formation or photoinduced electron transfer [33,40].

### 3.3. Enzymatic Cleavage Assay by Cells in Suspension

We investigated the enzymatic activity of live tumor cells towards the activatable imaging agents (**2** and **3**) using fluorescence spectroscopy. For this, SKOV-3 human ovary adenocarcinoma cells were chosen since they are known to express MMP-2/9 [35]. The imaging agents were added to the cells in suspension and the reaction was followed by measuring fluorescence emission spectra. The rationale behind these experiments is that the ratio between donor and acceptor emission intensity upon donor excitation provides a read-out that is independent of concentration [36]. As a result, this value provides a more quantitative read-out of the activation assay than the individual signal intensities. Hereby, it has to be taken into account that the ratio between donor and acceptor emission is influenced by both an increase in donor emission and a decrease in acceptor emission, meaning that the cleavage is effectively counted double in the donor/acceptor ratio.

**Figure 3:**
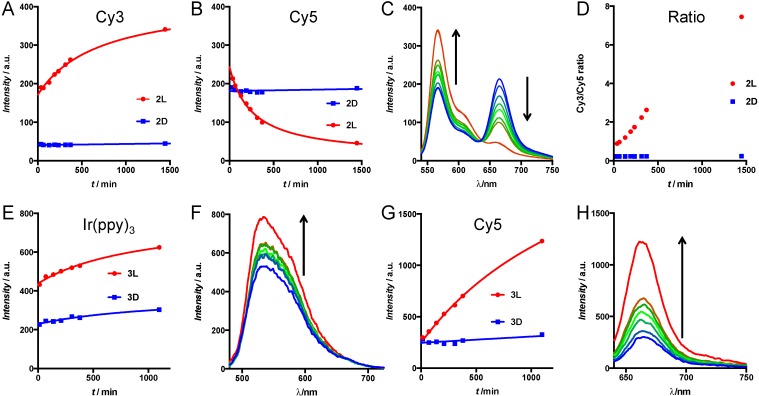
(**A**–**D**) Cleavage assay of **2L** and **2D** by MMP-expressing cells (**A**,**B**) Emission intensity from peptides **2L** (red circles) and **2D** (blue squares) of the donor peak (Cy3, excitation 525 nm, emission 566 nm) (**A**) and the FRET peak (Cy5, excitation 525 nm, emission 666 nm) (**B**) in time; (**C**) Variations in emission spectra (excitation 525 nm) of **2L** in time from blue (first time point) to red (last time point) and (**D**) the donor/acceptor ratio; (**E**–**H**) Cleavage assay of **3L** and **3D** by MMP-expressing cells; (**E**,**G**) Emission intensity changes from peptides **3L** (red circles) and **3D** (blue squares) of the peaks at 590 nm (**E**) and 666 nm (**G**) in time; (**F**,**H**) Change in emission of these peaks of **3L** in time from blue (first time point) to red (last time point) with excitation at 420 nm (**F**) or 625 nm (**H**). Arrows indicate change in time.

In **2L**, enzymatic cleavage resulted in a simultaneous increase in donor emission (566 nm) and decrease in acceptor emission (666 nm) (Figure 3A–C). In contrast, **2D** (the control) revealed no alterations when exposed to the enzymes, underlining that the D-amino acids do not get cleaved. This is exactly like expected for the enzymatically induced disruption of the FRET donor-acceptor pair in **2L**. In numbers, the intensity ratio Cy3/Cy5 of **2L** showed an eight-fold increase after cleavage, going from 0.9 to 7.7. In contrast, the compound with D-amino acids on the cleavage site (**2D**) only went from 0.22 to 0.27 after 24 h.

Similar to what we observed for peptides **2**, cleavage of lifetime imaging agents **3** resulted in an increase in donor-acceptor distance. The spin-orbit coupling that caused the quenching of the Cy5 emission is nullified after cleavage of peptide **3L**. This resulted in a four-fold increase of Cy5 emission in time, while the control compound **3D** only showed a 1.1-fold increase after 18 h (Figure 3E,F). The increase in Ir(ppy)_3_ emission was only minimal (Figure 3D). This can be explained by cellular uptake of the activated cell penetrating peptide that is formed after cleavage [15]. The combination of the many positive charges from the arginine residues with the lipophilicity of the Ir(ppy)_3_ creates a compound that is easily taken up by cells. This uptake effectively reduces Ir(ppy)_3_ concentration in the solution, which gives a flawed representation of activation. Apparently, the hydrophilicity and negative charges on Cy3 on **2L** partly prevented cellular uptake, since Cy3 activation was visible in solution. As a result, the ratio between donor and acceptor emission in Ir(ppy)_3_-Cy5 imaging agents cannot be used for signal quantification, since both donor and acceptor intensity increase in time.

### 3.4. Enzymatic Cleavage Assay Followed by Microscopy

**Figure 4:**
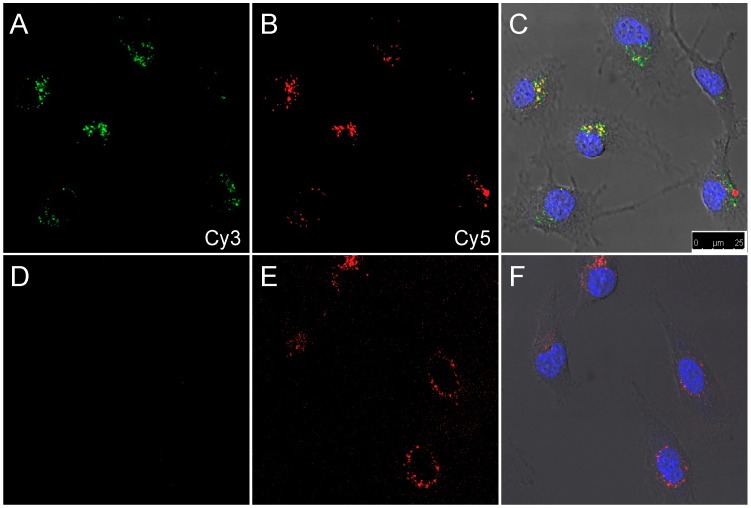
Confocal images of SKOV-3 cells after 24 h incubation with **2L** (**A**–**C**) or **2D** (**D**–**F**). (**A**,**D**) Cy3 channel in green; (**B**,**E**) Cy5 channel in red; (**D**,**F**) Overlay of differential interference contrast, nuclear stain (Hoechst 33342, blue), Cy3 (green), and Cy5 (red). Excitation and emission wavelengths are described in the Experimental Section.

After successful cleavage of the activatable imaging agents by cells in suspension, SKOV-3 cells were grown on a glass slide and incubated *in vitro* with **2L** and **2D**. Confocal microscopy was used to examine the cleavage activity after 24 h of incubation. Just as it was observed for the cleavage in suspension (Figure 3), peptide **2L** yielded an activated donor (Cy3) emission, which was not detected for **2D** (Figure 4B,E). The emission of Cy5 was used as a control, since the acceptor fluorescence upon direct acceptor excitation is not affected by the presence or absence of the Cy3 donor. Cy5 intensities of **2L** and **2D** were similar (Figure 4A,D).

**Figure 5:**
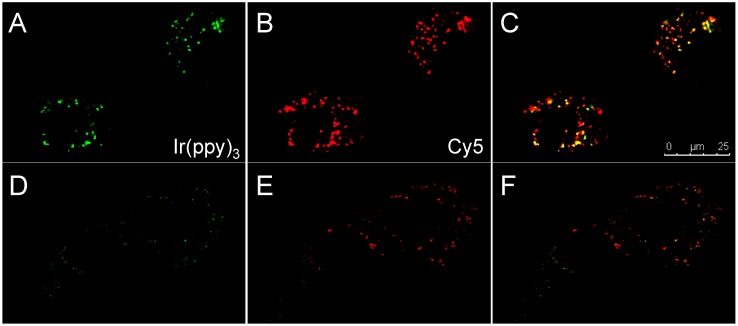
Confocal images of SKOV-3 cells after 24 h incubation with **3L** (**A**–**C**) or **3D** (**D**–**F**). (**A**,**D**) Ir(ppy)_3_ channel in green; (**B**,**E**) Cy5 channel in red; (**C**,**F**) Overlay of both channels. Yellow means colocalization of red and green.

The activation of compounds **3L** and **3D** was also tested using confocal microscopy. After 24 h incubation of SKOV-3 cells with either compound, signals of both Ir(ppy)_3_ and Cy5 were observed (Figure 5). Compound **3L** showed bright signals of both luminophores inside the cells, indicating successful cleavage of the activatable imaging agent **3L** *in vitro*. In contrast, in **3D** the emission intensities of both luminophores were very weak, possibly due to non-specific cleavage by other enzymes. Nuclear staining with Hoechst was not possible due to spectral overlap with Ir(ppy)_3_.

**Figure 6:**
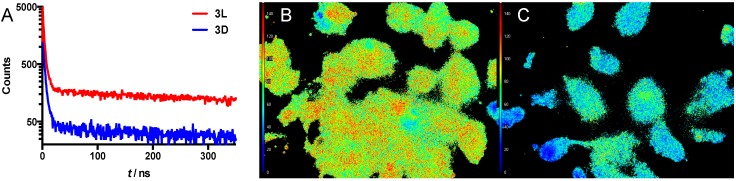
(**A**) Luminescence decay traces of a suspension of SKOV-3 cells after 24 h incubation with **3L** (red) or **3D** (blue); (**B**) FLIM image of cells incubated with **3L**; (**C**) FLIM image of cells incubated with **3D**. The scalebar on the left depicts τ, going from 0 ns (blue) to 150 ns (red).

Lifetimes were also used to measure the cleavage reaction in suspension. Since the initial signal intensities of the quenched starting point were very weak, long measurement times (>3 h per sample) were necessary to provide reliable lifetimes. This made dynamic measurements during the cleavage reaction impossible. Therefore, we measured the luminescence lifetime of **3L** and **3D** only after the cleavage reaction was complete (Figure 6A). Again, the uptake of the polycationic Ir(ppy)_3_ peptide caused low concentration of Ir(ppy)_3_ in solution, making lifetime measurements even more difficult. The only conclusion that can be drawn from these measurements is that there is relatively more long-lifetime activated Ir(ppy)_3_ present after incubation of **3L** compared to **3D** (Figure 6A). 

The potential of this new activatable lifetime imaging agent for Fluorescencence Lifetime Imaging Microscopy (FLIM) is shown in Figure 6B,C. After incubation of SKOV-3 cells with either compound **3L** or **3D** (24 h), the average lifetime of each pixel of the microscopy image was measured. From that, the average lifetime of the luminescence signal from cells was determined. For **3L**, the cleavable version, the average lifetime was 94 ns, while the control compound **3D** gave a substantially different average lifetime of 57 ns. These values are only slightly different from the respective lifetimes measured in solution, 98 ns (Ir(ppy)_3_) and 43ns (**3D**), respectively (see above). Based on the similarity in the spectroscopic measurements findings and the FLIM measurements, we feel confident that luminescence lifetime has provided a quantifiable readout for the MMP-2/9 induced activation of **3L**. This is the second time that we have shown the value of the Ir(ppy)_3_-Cy5 combination for activatable luminescence lifetime imaging, indicating that this combination has potential in different chemical formulations. Hence, it may also provide value in other—FRET based—designs for activatable imaging agents [41], thereby generating added value for the quantification of different disease related features. As alluded to in the introduction, a reduction in autofluorescence and thus non-specific background signal may be achieved using the concept of activatable lifetime imaging. In this concept, the sensitivity of enzymatically activated luminescence intensification can be strengthened further by enzymatically activated lifetime prolongation. This effect combined with a disease specific enzyme may help increase signal-to-background ratios between healthy and diseased tissue *in vitro* or in the future possibly also *in vivo*. The latter may help provide new imaging concepts in the field of luminescence-based interventional molecular imaging [42].

## 4. Conclusions

To conclude, FRET-based activatable imaging agents for the tumor-specific enzymes MMP-2/9 not only show potential for activatable luminescence imaging, in combination with Ir(ppy)_3_, this concept can now even be extended to the field of (microscopic) luminescence lifetime imaging. 

## Supplementary Files

Supplementary File 1
