# Supplementary material for: MMP-2/9-Specific Activatable Lifetime Imaging Agent
Source: Sensors (Basel). 2015 May 12;15(5):11076–91. doi: 10.3390/s150511076 (PMC4481940; doi:10.3390/s150511076)
Supplement: Supplementary File 1 [file sensors-15-11076-s001.pdf]

OPEN ACCESS

sensors

ISSN 1424-8220

www.mdpi.com/journal/sensors

Supplementary Information

MMP-2/9-Specific Activatable Lifetime Imaging Agent. *Sensors* 2015, *15*, 11076-11091

Marcus T.M. Rood [1], Marcel Raspe [2], Jan Bart ten Hove [1,3], Kees Jalink [2], Aldrik H. Velders [1,3] and Fijs W.B. van Leeuwen [1,3,*]

[1] Interventional Molecular Imaging Laboratory, Department of Radiology, Leiden University Medical Center, Leiden 2300RC, The Netherlands; E-Mails: m.t.m.rood@lumc.nl (M.T.M.R.); janbart.tenhove@wur.nl (J.B.H.); aldrik.velders@wur.nl (A.H.V.)

[2] Division of Cell Biology I, Netherlands Cancer Institute, Amsterdam 1066CX, The Netherlands; E-Mails: m.raspe@nki.nl (M.R.); k.jalink@nki.nl (K.J.)

[3] Laboratory of BioNanoTechnology, Wageningen University, Wageningen 6700EK, The Netherlands

* Author to whom correspondence should be addressed; E-Mail: f.w.b.van_leeuwen@lumc.nl; Tel.: +31-71-526-6029.

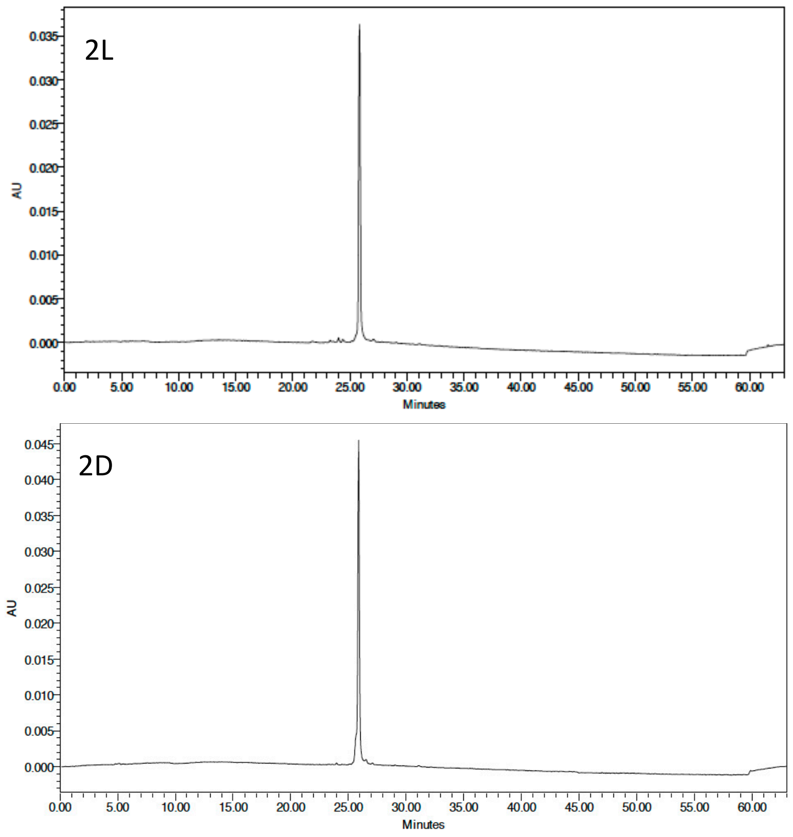

Figure S1. *Cont.*

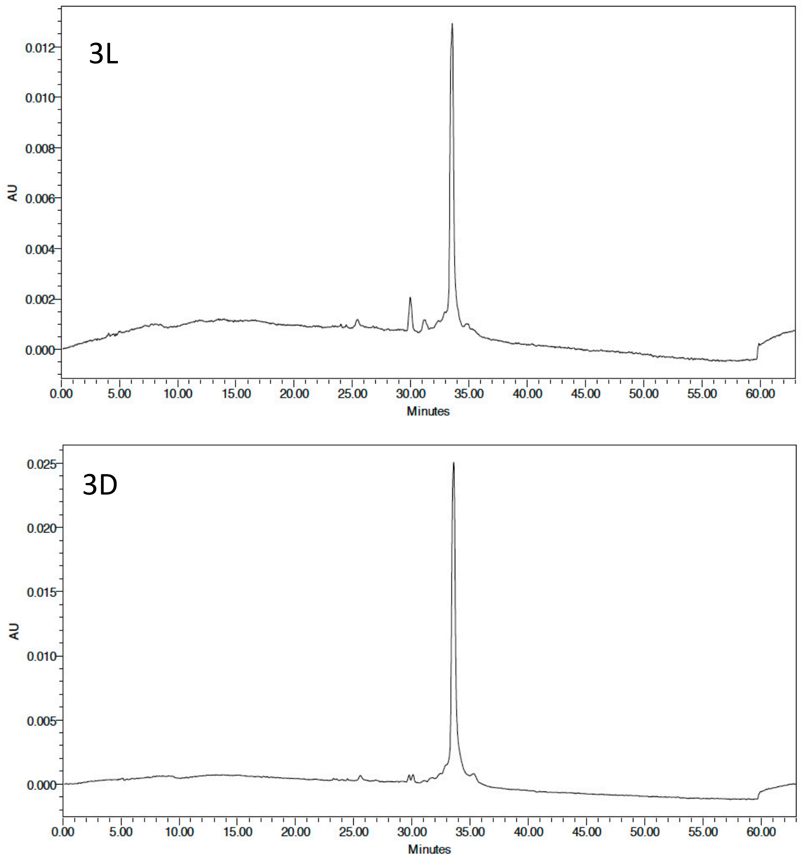

Figure S1. HPLC traces of the compounds used in this study.

© 2015 by the authors; licensee MDPI, Basel, Switzerland. This article is an open access article distributed under the terms and conditions of the Creative Commons Attribution license (http://creativecommons.org/licenses/by/4.0/).
